# Different Roles of Dioxin-Catabolic Plasmids in Growth, Biofilm Formation, and Metabolism of *Rhodococcus* sp. Strain p52

**DOI:** 10.3390/microorganisms12081700

**Published:** 2024-08-17

**Authors:** Xu Wang, Yanan Wu, Meng Chen, Changai Fu, Hangzhou Xu, Li Li

**Affiliations:** 1 Shandong Provincial Key Laboratory of Water Pollution Control and Resource Reuse, School of Environmental Science and Engineering, Shandong University, 72 Binhai Road, Qingdao 266237, China; 201820330@mail.sdu.edu.cn (X.W.); 201920359@mail.sdu.edu.cn (Y.W.); mengchen@sdu.edu.cn (M.C.); 201812484@mail.sdu.edu.cn (C.F.); 202099900205@sdu.edu.cn (H.X.); 2 Marine Genomics and Biotechnology Program, Institute of Marine Science and Technology, Shandong University, 72 Binhai Road, Qingdao 266237, China

**Keywords:** catabolic megaplasmids, dibenzofuran degradation, biofilm, transcriptional regulation, *Rhodococcus*

## Abstract

Microorganisms harbor catabolic plasmids to tackle refractory organic pollutants, which is crucial for bioremediation and ecosystem health. Understanding the impacts of plasmids on hosts provides insights into the behavior and adaptation of degrading bacteria in the environment. Here, we examined alterations in the physiological properties and gene expression profiles of *Rhodococcus* sp. strain p52 after losing two conjugative dioxin-catabolic megaplasmids (pDF01 and pDF02). The growth of strain p52 accelerated after pDF01 loss, while it decelerated after pDF02 loss. During dibenzofuran degradation, the expression levels of dibenzofuran catabolic genes on pDF01 were higher compared to those on pDF02; accordingly, pDF01 loss markedly slowed dibenzofuran degradation. It was suggested that pDF01 is more beneficial to strain p52 under dibenzofuran exposure. Moreover, plasmid loss decreased biofilm formation, especially after pDF02 loss. Transcriptome profiling revealed different pathways enriched in upregulated and downregulated genes after pDF01 and pDF02 loss, indicating different adaptation mechanisms. Based on the transcriptional activity variation, pDF01 played roles in transcription and anabolic processes, while pDF02 profoundly influenced energy production and cellular defense. This study enhances our knowledge of the impacts of degradative plasmids on native hosts and the adaptation mechanisms of hosts, contributing to the application of plasmid-mediated bioremediation in contaminated environments.

## 1. Introduction

In recent years, refractory organic pollutants, particularly those classified as emerging contaminants, have increasingly attracted concern, which has resulted from accelerated industrialization. Dioxins, a type of refractory organic compound with high toxicity, persistence, and bioaccumulation, pose a great threat to the environment and human health [1,2,3,4]. Bioremediation provides an eco-friendly approach for mitigating these pollutants via natural degradation facilitated by microorganisms. Plasmid-mediated bioremediation plays an essential role because plasmids encode genetic determinants that function in the catabolism of complex organic xenobiotics, facilitating the rapid adaptation of bacteria to diverse contaminants [5]. As an important vehicle of mobile genetic elements, conjugative plasmids are vital for the dissemination of degradation pathways through horizontal transfer, which plays an important role in bacterial survival and evolution in contaminated environments. The characterization of the effects of catabolic plasmids on the host offers insights into the behavior and fate of bacteria, paving the way for advances in environmental bioremediation.

Studies have focused on catabolic plasmids that confer the ability of hosts to degrade complex pollutants, such as naphthalene, carbazole, polychlorinated biphenyl, and dioxin-like compounds, thereby deepening our knowledge of genetic mechanisms underlying the degradation of various pollutants [6,7,8,9]. Several different evolved phenol degradation plasmids are detected in *Pseudomonas* strains that have been bioaugmented in a phenol-polluted environment [10]. However, the impact of plasmids on hosts extends beyond the capabilities conferred by plasmid-encoded genes.

Plasmids profoundly impact multiple aspects of bacterial physiology involving growth rate, biofilm formation, motility, as well as the gene expression of diverse bacterial phenotypes [11]. Plasmids engage in complex cross-talk with the host chromosome after acquisition [12], which leads to significant alterations in the gene expression profiles of host chromosomes, reprogramming metabolic pathways [13]. For instance, global regulator homologs commonly encoded by plasmids can manipulate the behaviors of bacteria via translational regulatory cross-talk [14]. Previous studies revealed that plasmid carriage disrupts DNA replication, transcription, and translation, and causes alterations in ATP synthesis [14,15,16]. The acquisition of plasmids incurred fitness costs on hosts, often manifested as metabolic burden that may result in the growth retardation of the strain [17], as compromised chromosome replication [13] and transient growth delay [18] were previously observed. In addition, natural conjugative plasmids were shown to induce the development of bacterial biofilms [19], a trait crucial for cells to survive under environmental stresses [20] and significant in the degradation of pollutants [21,22]. Virulence plasmids enhanced biofilm formation in *Klebsiella quasipneumoniae* [23], while the curing of RepA-I-type plasmids led to almost complete loss of motility and biofilm formation capabilities [24]. Transcriptional regulators encoded by virulence plasmids can significantly contribute to the reprogramming of the global transcription of *Rhodococcus equi* [25]. Genes whose expression is altered resulting from plasmids account for 8% of the total gene pool of *Bifidobacterium breve* and are involved in multiple pathways, including biofilm production, carbohydrate transport and metabolism, and exopolysaccharide biosynthesis [26]. The presence of the carbazole-catabolic plasmid pCAR1 induces distinct gene transcription profiles across different strains [27]. The plasmids affect bacterial behaviors through altering the expression of crucial phenotypes; however, these processes remain poorly understood [14]. The impacts of natural conjugative plasmids on gene functions and transcriptomes, particularly those involving multiple catabolic megaplasmids in native hosts, are rarely studied.

In this study, in contrast to the study focused on plasmid acquisition, we examined how the loss of two catabolic megaplasmids (pDF01 and pDF02) impacts physiologic properties, including phenotypic changes and cellular function, in the native bacterial host *Rhodococcus* sp. strain p52. Strain p52, a Gram-positive bacterium with high DNA G+C content, is rod-shaped and lacks pili. Strain p52 is capable of utilizing a variety of refractory organic pollutants as the sole carbon sources, such as dibenzofuran, dibenzo-*p*-dioxin, naphthalene, fluorene, and carbazole. These two catabolic plasmids contain gene clusters that encode two distinct angular dioxygenases (DfdA and DbfA), which are involved in the initial dihydroxylation of dibenzofuran [28]. Strain p52, harboring two conjugative catabolic plasmids, has been demonstrated to be a competent candidate for the plasmid-mediated bioaugmentation of activated sludge reactors during the removal of dibenzofuran [29]. Strain p52 can produce biosurfactants and excrete extracellular polymeric substances when bioaugmented in activated sludge reactors with the presence of non-aqueous liquids, thus promoting dibenzofuran removal [30]. The cross-feeding can occur between strain p52 and the co-cultivated bacterium and enhances the growth of strain p52 [31]. However, the distinct effects of the plasmids on strain p52 physiology, as well as their specific roles during pollutant degradation, remain poorly understood. This study aims to evaluate the roles of pDF01 and pDF02 in strain p52. To achieve this, we investigated alterations in the functions and gene expression profiles of *Rhodococcus* sp. strain p52 after the plasmid loss. Growth monitoring, biofilm formation assay, and dibenzofuran degradation experiment were conducted after plasmid loss. Through growth analysis, the distinct impacts on the growth of the plasmids were determined. Furthermore, the expression levels of dibenzofuran catabolic genes on pDF01 and pDF02 were, respectively, detected to determine their priority during the degradation process. Transcriptomic analysis was conducted to elucidate the impacts on pathways enrichment in strain p52, which revealed the adaptation mechanisms in response to different plasmid loss. This study provides insights into the impacts of different catabolic plasmids on the same native host and the regulatory strategies of the host in response to the plasmid loss. The knowledge is crucial for understanding the full spectrum of plasmid functions and roles in bacterial survival and evolution and, ultimately, paving a way in bioremediation for refractory organic pollution.

## 2. Materials and Methods

### 2.1. Chemicals and Bacterial Culture

The bacterium used in this study was *Rhodococcus* sp. strain p52, which was isolated from oil-polluted soil and deposited in the China Center for Type Culture Collection (no. M2011181). Strain p52 was designated as a *Rhodococcus* sp. based on the 16S rRNA gene sequence [28]. The genome sequence of strain p52 was deposited in GenBank under the assembly accession GCA_000763325.2.

The chemicals employed in this study were of analytical grade or molecular biology grade. Dibenzofuran, used as a model compound for dioxin biodegradation, was purchased from Sigma–Aldrich (Shanghai, China). Other chemicals were purchased from Sangon Biotech (Shanghai, China). Luria–Bertani (LB) medium [32] and carbon-free mineral medium (CFMM) [33] were prepared as previously described. All the bacteria were cultivated at 30 °C and the strains in liquid media were continuously shaken at 180 rpm.

### 2.2. Plasmid Curing Experiment

Plasmid curing experiments were conducted with a temperature-based method [34]. Briefly, strain p52 was grown overnight in the LB broth. Subsequently, a 1% (*v*/*v*) inoculum was transferred into the fresh LB broth and cultivated at 42 °C. Sequential transfers were carried out every 24 h. During each transfer, the culture was streaked onto LB plates, and then a single colony was randomly selected for PCR. The colony PCR was conducted to detect the presence of dioxygenase genes, *dfdA* on pDF01 and *dbfA* on pDF02, to identify the plasmid presence/absence. The primer sequences are listed in Table S1. The PCR products were checked by electrophoresis and further by sequencing analysis if needed. Moreover, plasmid extraction was conducted according to Ka et al. [35] to confirm the loss of plasmids in the strain. The pDF01-cured strain p52 (marked as p52 (pDF01^−^, pDF02) in this study), and both pDF01- and pDF02-cured strain p52 (marked as p52 (pDF01^−^, pDF02^−^) hereafter) were acquired and preserved in our laboratory.

### 2.3. Cell Growth Rate Analysis

Cell growth rate analysis was conducted by the optical density (OD_600nm_) measurement of the strains during growth. Individual colonies of strain p52, p52 (pDF01^−^, pDF02), and p52 (pDF01^−^, pDF02^−^) were cultivated in the LB broth until OD_600_ = 1.0. Then, 1% (*v*/*v*) of each culture was transferred to fresh LB broth, and OD_600_ was measured every 2 h using a UV–Vis spectrophotometer (HACH, Düsseldorf, Germany). The data were fitted using a logistic growth model with the Levenberg–Marquardt algorithm and the growth rate is the first derivative of the fitted curve. The specific growth rates were calculated using the periods of logarithmic growth, which was calculated by ln (*N*_*t*2_/*N*_*t*1_)/(*t*_2_ − *t*_1_), where *N_t_* is the cell numbers at time *t*. Three biological replicates were conducted for each sample. To determine the growth of strains under the dibenzofuran environment, the plate spreading method was used to determine the number of single colonies per milliliter (CFU/mL).

### 2.4. Scanning Electron Microscope (SEM)

SEM observation was conducted to detect the morphological differences after plasmid loss. Briefly, the cells were collected and washed with phosphate-buffered saline (PBS) twice, and fixed with 500 µL of 2.50% (*v*/*v*) glutaraldehyde for 4 h at 4 °C, and rinsed with 0.1 M phosphate buffer; then they were fixed with 1% osmium tetroxide for 90 min and rinsed again with 0.1 M phosphate buffer. Sequentially, the cells were dehydrated through an ethanol gradient series (each step for 10 min). After replacing twice with isoamyl acetate and drying using a critical point dryer, the samples were coated with gold using an IB-3 ion sputtering device (Eiko, Tokyo, Japan). Finally, the samples were observed using a VEGA3 SEM (TESCAN, Brno, Czech Republic).

### 2.5. Flow Cytometric Analysis

Flow cytometry was used to analyze the size, shape, or granularity of the cells at the population level. The effects of plasmid bearing on the morphology of strain p52 were investigated. First, individual colonies of strain p52, p52 (pDF01^−^, pDF02), and p52 (pDF01^−^, pDF02^−^) were inoculated into LB broth. The cells at the early, mid, and late logarithmic phases (OD_600_ = 0.5, 1, and 1.7) were harvested at a cell density of approximately 10^7^ CFU/mL. The morphological variations among the cells were assessed using specific flow cytometry parameters. Relative cell size was estimated by the forward scatter (FSC) area; cellular granularity was determined by the intensity of side scatter (SSC) [36]; and cell shape was assessed through the aspect ratio, which was calculated as the ratio of the major axis to the minor axis for each cell [37]. For each sample, 10,000 cells were analyzed by flow cytometry (Amnis ImageStreamX Mark II, Seattle, WA, USA), and the data were analyzed using the IDEAS software (version 6.0).

### 2.6. Degradation Experiments

Strain p52 exhibited high tolerance to dibenzofuran, and growth on different concentrations of dibenzofuran has been tested (Supplementary text, Figure S1). Based on this, the biodegradation experiment was conducted. The strains p52, p52 (pDF01^−^, pDF02), and p52 (pDF01^−^, pDF02^−^) were inoculated into 50 mL of CFMM supplemented with 100 mg/L dibenzofuran in 250 mL Erlenmeyer flasks. First, the clones were precultured in the LB medium to the logarithmic phase, and then the cells were collected by centrifugation (6000 rpm, 8 min, and 4 °C) and washed twice with PBS. The cells were resuspended in CFMM to an OD_600_ of approximately 1 (10^7^ CFU/mL), and 4% of the suspended cells were inoculated into CFMM with 100 mg/L dibenzofuran, while the noninoculated sample was used as a negative control. The cultures of the strains were incubated at 30 °C with shaking at 180 rpm. The concentration of residual dibenzofuran in the culture was determined by gas chromatography as previously described [28]. Three biological replicates were conducted for each sample.

### 2.7. Biofilm Measurement

To investigate the impact of plasmids on the biofilm formation of strain p52, the biofilm contents of the plasmid-bearing clones and the plasmid-free clones were measured and compared under various nutritional conditions. The biofilm density was determined by the crystal violet staining method [38]. Strain p52 (5%, *v/v*) was inoculated into 96-well plates containing 200 µL of LB, 55.5 mM glucose, and 166.5 mM sodium acetate as nutrients, respectively, and cultivated at 30 °C in static culture. After incubation for 36 h, the planktonic bacteria were discarded, and the plates were washed three times with CFMM to remove the unattached cells. Then, 200 μL of methanol was added to immobilize the biofilms for 15 min, the methanol was discarded, and the plates were air-dried at room temperature. Subsequently, 200 μL of 0.1% crystal violet staining solution was added for 30 min, after which the crystal violet was discarded, and the wells were rinsed three times with sterile water before air drying at room temperature. Finally, the crystal violet that bound to the biofilm was dissolved in 200 μL of acetic acid (33%). Medium without any bacteria was used as a negative control. All the samples were processed in at least ten wells. The biofilm density was evaluated by OD_595_ using a microplate reader (Thermo Fisher Scientific, Waltham, MA, USA) and was calculated by subtracting the absorbance of the control from that of the experimental groups.

### 2.8. Reverse Transcription Quantitative PCR (RT–qPCR)

RT–qPCR was conducted to evaluate the gene expression levels. The detections involved the expression of catabolic genes in strain p52 during dibenzofuran degradation, as well as genes related to biofilm formation following plasmid loss. Total RNA extraction was performed using a *SteadyPure* Universal RNA Extraction Kit (Accurate Biotechnology Co., Ltd., Hunan, China) according to the manufacturer’s instructions. The RNA quality and quantity were determined by 1% agarose gel electrophoresis and NanoDrop 2000 spectrophotometry. The gDNA was purified and cDNA was synthesized using an *Evo M-MLV* RT mix kit (Accurate Biotechnology Co., Ltd., Hunan, China), followed by qPCR using a SYBR Green Pro Taq HS qPCR kit (Accurate Biotechnology Co., Ltd., Hunan, China). The qPCR was performed on a Q1000 + RT–qPCR system (LongGene Instruments, Hangzhou, China). For all the qPCR, an initial denaturation step was performed for 3 min at 95 °C, followed by 40 cycles of denaturation at 95 °C for 5 s and annealing/extension for 30 s at 58 °C. The *rpoB* gene of strain p52 was used as a reference gene, and the relative gene expression levels were calculated as the log_2_(fold change) [39]. The primers used for the qPCR are listed in Table S1.

### 2.9. Transcriptome Analysis

The clones of strains p52, p52 (pDF01^−^, pDF02), and p52 (pDF01^−^, pDF02^−^) were cultivated in the LB broth (30 ℃, 180 rpm) overnight to the logarithmic phase (OD_600_ = 1). The cells were harvested by centrifugation at 6000 rpm for 8 min at 4 °C. Total RNA was extracted following the manufacturer’s instructions for the TRIzol^®^ Reagent (Invitrogen, Carlsbad, CA, USA). RNA purity and concentration were assessed using a NanoDrop 2000 spectrophotometer (NanoDrop Technologies, Wilmington, DE, USA), and the RNA integrity number (RIN) was determined using an Agilent 5300 (Agilent, Santa Clara, CA, USA). Sequencing was performed on the Illumina HiSeq X Ten platform (Majorbio Bio-Pharm Technology Co., Ltd., Shanghai, China). Three biological replicates were performed for each sample. The clean reads were mapped to the reference genome of the complete genome of *Rhodococcus* sp. strain p52 (GenBank assembly accession No. GCA_000763325.2). A cluster heatmap was constructed to represent the gene expression levels through normalizing the data. Initially, logarithmic transformation was applied to (TPM + 1) (TPM: transcripts per million), followed by Z-score standardization. Differentially expressed genes (DEGs) were analyzed by DESeq2 based on the negative binomial distribution, with the parameter settings meeting that of an absolute log_2_(fold change) of TPM being greater than 1 and p-adjust being less than 0.05. The functional categories of the genes were annotated based on the clusters of orthologous groups (COG). A Gene Ontology (GO) enrichment analysis of DEGs was performed using Goatools and Fisher’s test. The GO functions were considered significantly enriched when the *p*-adjust value was less than 0.05.

### 2.10. Statistical Analysis

The data are expressed as the mean ± standard deviation (SD). The significant differences between the treatments (clones of strain p52, p52 (pDF01^−^, pDF02), and p52 (pDF01^−^, pDF02^−^)) and control were assessed by the one-way analysis of variance. The homogeneity of variances was determined by Levene’s test for equal variances. For the groups with equal variances, Fisher’s least significant difference (LSD) test was used for post hoc testing, and Tamhane’s T2 test was employed for the groups with unequal variances. Statistical significance was determined at the 0.05 level (*) or 0.01 level (**) for all the tests.

## 3. Results and Discussion

### 3.1. Effects of pDF01 and pDF02 on Cell Growth

**Figure 1:**
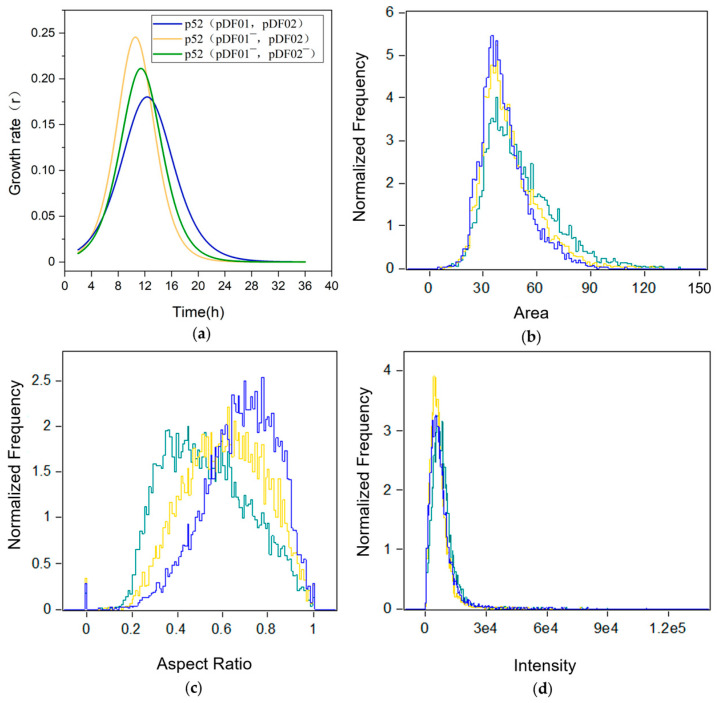
The impact of plasmid loss on the growth of strain p52. (**a**) Cell growth rate (*r*) throughout the growth process. (**b**) Morphological analysis of the clones of strain p52 at the mid-logarithmic phase (OD_600_ = 1) at the population level using flow cytometry. The normalized frequency of the forward scatter area was used to estimate the cell size. (**c**) The normalized frequency of the aspect ratio was used to estimate the cell shape. (**d**) The normalized frequency of the intensity of side scatter was used to estimate cellular granularity. The clones of strains p52, p52 (pDF01^−^, pDF02), and p52 (pDF01^−^, pDF02^−^) are represented by blue, yellow, and green lines, respectively.

Investigating the strain growth difference is essential, as it provides fundamental insights into how the plasmid affects the survival and reproduction of bacteria [18]. The plasmid-cured clones were obtained after the plasmid curing experiment (Figure S2). To explore the influence of catabolic plasmids on cell growth, the growth rate *r*, specific growth rate *μ*, and morphology of strains p52, p52 (pDF01^−^, pDF02), and p52 (pDF01^−^, pDF02^−^) were evaluated without selective pressure. After pDF01 loss, compared to the strain p52, p52 (pDF01^−^, pDF02) grew faster during the logarithmic phase (Figure 1a), which revealed that the loss of pDF01 leads to an increase in the growth of strain p52. However, compared to p52 (pDF01^−^, pDF02), p52 (pDF01^−^, pDF02^−^) grew more slowly during the logarithmic phase (Figure 1a), which revealed that the subsequent loss of pDF02 slowed down the growth of strain p52. The specific growth rate *μ* for p52 (pDF01^−^, pDF02) at the logarithmic phase (from approximately the 6th hour to the 12th hour) was greater than that of p52 (pDF01^−^, pDF02^−^), while strain p52 showed the lowest value (Figure S3). This result suggested that pDF01 carriage was unfavorable to strain p52 growth, in contrast to that of pDF02. Generally, the carriage of plasmids capable of degrading certain pollutants could impose a burden on the cells when the target pollutants were absent. The burden may arise from the metabolism relating to plasmid function, which requires resources provided by the hosts [40]. Previously, the carriage of carbazole-degradative megaplasmid pCAR1 has reduced the growth rates of the host in the early logarithmic phase, and one of the factors is ascribable to the expression of the carbazole degradative enzymes on the plasmid [41]. However, some natural plasmids may confer benefits to the host, even in the absence of selective pressure [42].

The SEM images showed no marked differences between plasmid-bearing and plasmid-free clones during the logarithmic growth phase (Figure S4). Considering that the SEM observations are based on selected individual cells, flow cytometry was conducted at the early, middle, and late logarithmic growth phases at the population level. The distribution of morphological characteristics was quantified using the normalized frequency to evaluate the disparities across the three distinct strains. The results showed that there were small morphological differences among all three strains during the early and late logarithmic phases (Figure S5), and the differences were relatively great during the mid-logarithmic phase. The peak in the normalized frequency distribution for p52 (pDF01^−^, pDF02^−^) showed a notable decrease compared to the other two strains, suggesting that cell size is significantly affected when both the plasmids are lost; in contrast, the loss of only pDF01 has an unnoticeable impact (Figure 1b). The most pronounced effects of plasmid loss were observed on the cellular aspect ratio. Specifically, a marked decrease was observed at p52 (pDF01^−^, pDF02^−^) (Figure 1c), reflecting decreased circularity due to the absence of the two plasmids. The frequency of SSC in p52 (pDF01^−^, pDF02) exhibited a slightly greater peak (Figure 1d), suggesting that the loss of pDF01 has a slight impact on cellular granularity.

Based on the genomic annotation at NCBI, genes related to cell morphology were identified in strain p52, which encodes the cell division proteins FtsZ, FtsW, and FtsI [43] and the cell wall synthesis protein DivIVA [44]. Transcriptomic analysis revealed that the loss of pDF01 resulted in slight changes in the expression levels of these genes. However, when both plasmids were lost, the gene encoding FtsW was significantly upregulated (Figure S6). FtsW is a peptidoglycan polymerase that helps produce septal peptidoglycan during cell division, and the peptidoglycan cell wall is crucial for bacterial morphogenesis and survival [45]. The results indicated that the loss of plasmids, especially pDF02, had a relatively great impact on the cell size and shape of strain p52 during the mid-logarithmic phase. This effect may depend on the differences in cell growth rates caused by plasmid loss, as growth delay correlating to plasmid carriage primarily occurred during the logarithmic phase [18]. The significant change in the expression level of *ftsW* suggested that plasmid loss plays an essential role in the cell division and growth of strain p52.

### 3.2. Distinguishing Roles of pDF01 and pDF02 in Dibenzofuran Degradation

**Figure 2:**
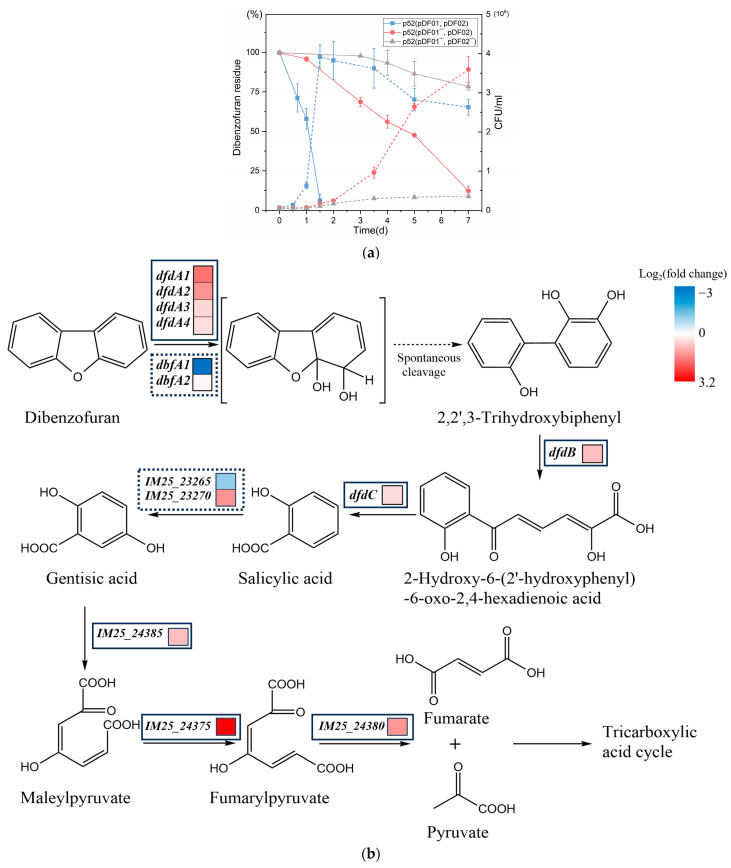
Dibenzofuran degradation along with the growth of strain p52, p52 (pDF01^−^, pDF02), and p52 (pDF01^−^, pDF02^−^) and relative expression levels of catabolic genes on pDF01 and pDF02 during degradation. (**a**) Growth of strain p52, p52 (pDF01^−^, pDF02), and p52 (pDF01^−^, pDF02^−^) and the residue dibenzofuran. The solid line represents the residual dibenzofuran, while the dashed line represents the strain growth by colony count. (**b**) Relative expression levels of related catabolic genes for dibenzofuran degradation pathway in strain p52, including the genes encoding two salicylate 5-hydroxylases (IM25_23265 and IM25_23270), a gentisate 1,2- dioxygenase (IM25_24385), a maleylpyruvate isomerase (IM25_24375), and a fumarylacetoacetate hydrolase family protein (IM25_24380). The solid frames indicate the genes on pDF01, while the dashed lines indicate the genes on pDF02.

Comparing the pollutant degradation abilities of strains with and without plasmids can reveal the contribution of plasmids to cells in handling environmental contaminants, which is crucial for the development and optimization of bioremediation technologies. The intermediates of dibenzofuran metabolism have been identified, and the degradation pathway has been deduced [28,31] as shown in Figure 2. In the presence of dibenzofuran as the sole carbon source, 6.2% residual dibenzofuran was detected at 36 h when the plasmids were present (Figure 2a). For p52 (pDF01^−^, pDF02), there was a notable increase in the duration of dibenzofuran degradation. On the 3rd day, 68.7% of the dibenzofuran residue was still present. By the 7th day, 12.3% of the residue remained. The cell density of strain p52 in the culture rapidly increased within 36 h when bearing plasmids, and reached the maximum before a gradual decline. However, after the loss of pDF01, the density of strain p52 gradually increased after 2 d (Figure 2a). The results demonstrated that the loss of pDF01 significantly reduced the cell growth in the dibenzofuran-exposed environment and the efficiency of dibenzofuran degradation, suggesting that pDF01 primarily functions in the degradation process. The relative expression levels of catabolic genes during dibenzofuran degradation were measured by RT–qPCR. The strain p52 at the initial time was used as the control group, and the experimental group was chosen at the 30th hour during the degradation process. The results demonstrated that the expression levels of the catabolic genes *dfdA1*, *dfdA2*, *dfdA3*, and *dfdA4* within the *dfdA* gene cluster on pDF01 were significantly greater than those of *dbfA1* and *dbfA2* within the *dbfA* gene cluster on pDF02 (Figure 2b). Furthermore, most of the genes involved in the entire pathways of dibenzofuran degradation are encoded by pDF01. The higher expression levels of these genes further highlighted the crucial role of pDF01 in the degradation process.

There are several reports on the Gram-positive strains that coharbor the *dfdA* and *dbfA* gene clusters. The expression of the two gene clusters was detected during the degradation of dibenzofuran by *Rhodococcus* strain HA01 [46]. With regard to *Terrabacter* sp. DBF63, the reverse transcription-PCR of *dbfA1A2* on the plasmid pDBF1 was performed to elucidate its function in dibenzofuran degradation [47]. The extremely high expression of *dfdA1* compared to *dbfA1* after growth on dibenzofuran was detected [48]. The results suggested that although both gene clusters encoded by strain p52 were involved in dibenzofuran degradation, compared to pDF02, pDF01 played a more predominant role in the initial ring-dihydroxylation of dibenzofuran. Therefore, plasmids conferred advantages to cells in the presence of dibenzofuran, rather than a burden to the cells without selective pressure. When dibenzofuran was used as the sole carbon source for strains p52, pDF01 and pDF02, both of which encode angular dioxygenase for dibenzofuran dihydroxylation, may compete. When plasmids compete for the same beneficial function, only the most advantageous plasmid is retained, which is analogous to the competitive exclusion observed in ecological communities [49]. pDF02 was defeated during competition for the initial dibenzofuran dihydroxylation; however, it was retained due to its indispensable role in the subsequent degradation steps. Although pDF02 carriage was favorable to the strain p52 growth in contrast to that of pDF01 under non-selection pressure, this situation is not static. A similar situation was observed with two mercury resistance plasmids; although the advantageous plasmid outcompeted the other in environments without mercury, the competitive hierarchy was reversed under mercury environments [50]. Overall, pDF01 was beneficial to strain p52 under dibenzofuran exposure though unfavorable to strain p52 growth without selection pressure.

### 3.3. pDF02 Loss Resulted in Reduced Biofilm Formation

**Figure 3:**
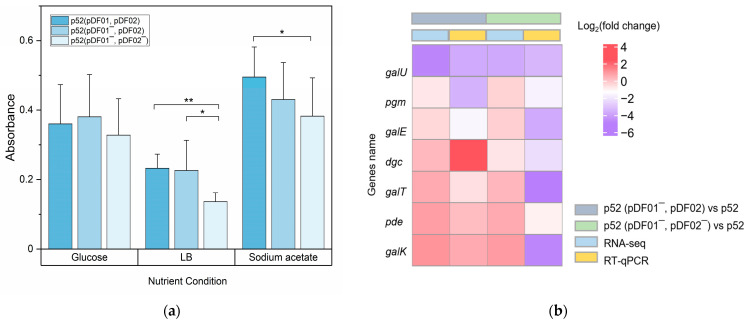
Biofilm formation and related relative gene expression in strains p52, p52 (pDF01^−^, pDF02), and p52 (pDF01^−^, pDF02^−^). (**a**) Biofilm of the strains p52, p52 (pDF01^−^, pDF02), and p52 (pDF01^−^, pDF02^−^) when grown in LB, and mineral mediums with sodium acetate or glucose as carbon sources. Significant differences between different nutritional conditions are indicated by asterisks at *p* < 0.05 (*) and *p* < 0.01 (**). (**b**) Relative expression levels of the genes involved in biofilm formation according to RNA-seq and RT–qPCR in the two comparison groups: p52 (pDF01^−^, pDF02) vs. p52 and p52 (pDF01^−^, pDF02^−^) vs. p52 (pDF01^−^, pDF02).

Biofilm formation capacity is linked to interactions within bacterial communities and affects bacteria defense mechanisms [51]. Studying changes in biofilm formation can highlight the contribution of plasmids to the behavior of bacterial populations. To explore the effects of plasmids on biofilm formation, the biofilms of strain p52, p52 (pDF01^−^, pDF02), and p52 (pDF01^−^, pDF02^−^) were measured under three different nutritional conditions. The results indicated that the greatest amount of biofilm was produced under sodium acetate as carbon source conditions, while the least amount was produced under LB-nourishing conditions (Figure 3a). After the loss of pDF01, little change in biofilm formation was observed. Notably, for p52 (pDF01^−^, pDF02^−^), biofilm formation markedly decreased under all three nutritional conditions, suggesting that pDF02 was related to biofilm formation and the loss of pDF02 was unfavorable for biofilm formation.

Changes in the expression levels of genes involved in biofilm formation were analyzed via the transcriptomic analysis and RT–qPCR after plasmid loss. The genes *galU*, *galK*, *galE*, *galT* [52,53], *pgm* [54], *dgc*, and *pde* [55] were identified on the chromosome of strain p52. The transcriptome results showed that after pDF01 loss, genes (*galU*, IM25_02690) encoding UDP-glucose pyrophosphorylase were downregulated, and a gene (*galK*, IM25_02380) encoding galactokinase was upregulated; in contrast, other related genes, such as *galT*, *galE*, *galK*, *pgm*, *dgc*, and *pde*, did not exhibit significant changes (Figure 3b). Compared to that in strain p52, only *galU* was significantly downregulated when both plasmids were lost. RT–qPCR showed that after pDF01 loss, *dgc* was significantly upregulated, while *galU*, *pgm,* and *galE* were downregulated. After the subsequent loss of pDF02, all the detected genes except *pde* were significantly downregulated, which suggested that the loss of pDF02 has a detrimental effect on biofilm formation.

The results demonstrated that biofilm formation decreased after plasmid loss. It was indicated that the loss of catabolic plasmid, especially pDF02, may affect the transcriptional regulation of chromosomal genes involved in biofilm formation. The marginal change in biofilm formation for p52 (pDF01^−^, pDF02) may be attributed to the simultaneous upregulation and downregulation of biofilm-related genes. However, the marked decrease in biofilm formation observed upon the loss of both plasmids may primarily result from the downregulation of multiple genes, especially *galU*. *galU* plays a crucial role in the synthesis of UDP-glucose that is necessary for the biosynthesis of extracellular polysaccharides and capsular polysaccharides [56]. This finding suggested that plasmid carriage, especially pDF02, facilitated biofilm formation.

### 3.4. Metabolic Adaptation in Response to the pDF01 and pDF02 Loss

**Figure 4:**
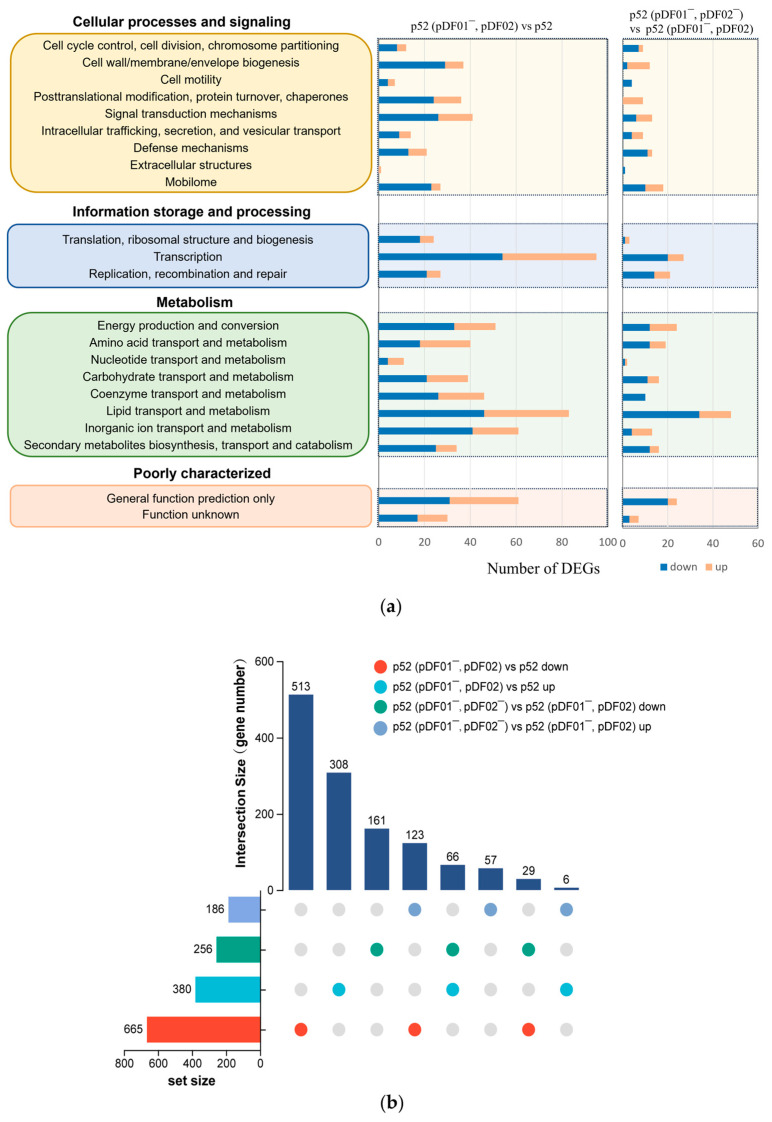
Statistics of differentially expressed genes (DEGs). (**a**) COG category statistics of DEGs. The functional description of each COG category is grouped on the left. The number of DEGs including up- and downregulation within each category is given in the horizontal bars. COG category statistics between p52 (pDF01^−^, pDF02) vs. p52, and p52 (pDF01^−^, pDF02^−^) vs. p52 (pDF01^−^, pDF02) are, respectively, listed on the right. (**b**) UpSet Venn diagram of the up- and downregulated DEGs in the four comparison groups (labeled on the upper right of the figure in colored circles). The intersection size of the top section represents the number of genes shared among the comparison groups. The set size at the bottom left section of the figure represents the total number of DEGs in each corresponding group. The solid circles in the bottom right matrix colored corresponding to the comparison groups indicate genes that are present in multiple comparison groups simultaneously. The number of their shared genes is shown by the stand column in the top section.

Transcriptome analysis is an essential way to understand how plasmid loss affects genome-wide gene expression and regulation mechanisms, offering insights into bacterial adaptations at the molecular level. Prior to transcriptome analysis, the annotation of the plasmid sequences was accomplished based on the reference genome after a complete genome sequencing analysis of strain p52, which has been depicted in plasmid maps (Figure S7). pDF01 contains numerous genes related to transport and metabolism, while pDF02 harbors many genes associated with replication, recombination, and repair. To determine the impacts of the two catabolic plasmids on strain p52, the gene transcription levels between plasmid-bearing clones and plasmid-free clones were genome-wide analyzed. Strains p52 and p52 (pDF01^−^, pDF02) were compared to explore the effects of pDF01, while the effects of pDF02 were determined by comparing p52 (pDF01^−^, pDF02) and p52 (pDF01^−^, pDF02^−^). A cluster heatmap was constructed to visualize the gene expression levels (Figure S8). The results indicated that upon plasmid loss, the number of downregulated genes exceeded that of upregulated genes. Compared to the subsequent loss of pDF02, the loss of pDF01 triggered a more extensive alteration in the expression profile based on the number of DEGs annotated by COG functional category assignments (Figure 4a). Among all the COG classifications, the loss of pDF01 resulted in the greatest number of DEGs within the transcription category, which had the greatest number of upregulated and downregulated genes. As previously demonstrated, the altered expression of regulatory genes caused by plasmids can lead to changes in the expression levels of numerous genes across the genome [25]. The results also showed that the number of DEGs associated with the metabolism category was impressively high. In a previous study, plasmid acquisition led to DEGs involved in bacterial functions, such as metabolism, translation and transcription, respiration, signaling, motility, secretion systems, the tricarboxylic acid cycle, and iron acquisition [57]. As reported, metabolism is the most commonly enriched function among DEGs, even with the acquisition of different plasmids [13]. Studies have also shown that the altered metabolic pathways generally involved amino acid metabolism, energy production, lipid transport, carbohydrates, and nitrogen [13,58]. The present study showed that the DEGs in the metabolism category mainly involved lipid transport and metabolism, inorganic ion transport and metabolism, energy production and conversion, and coenzyme transport and metabolism. Notably, plasmids principally burden hosts through transcription and metabolism [11]. The marked impact of pDF01 loss on DEGs in the transcription and metabolism categories suggested that pDF01 plays an important role in these processes.

To further explore the DEGs among the comparison groups, an UpSet Venn diagram was constructed to show the intersections and uniqueness of the DEGs in the different comparison groups (Figure 4b). All four comparison groups contained a great number of unique DEGs, which further reflects the distinct expression profiles resulting from the loss of pDF01 and pDF02. Among the intersections of DEGs in different comparison groups, the number of genes that were downregulated after the loss of pDF01 but upregulated after the subsequent loss of pDF02 was the largest (123); however, most of the proteins encoded by these genes are hypothetical proteins, while others are involved in transcriptional regulators, transferases, and genetic information processing, such as transposases and integrases. The genes whose expression was upregulated after the loss of pDF01 but downregulated following the subsequent loss of pDF02 were the second-largest group (66). The genes mainly encode oxidoreductases, transporters, and hypothetical proteins. The results suggested that a differential regulatory mechanism occurred and the specific genes expressed differently in response to these two distinct conditions (the loss of pDF01 or pDF02).

#### 3.4.1. pDF01 Played Roles in Cellular Metabolism and Transcription

**Figure 5:**
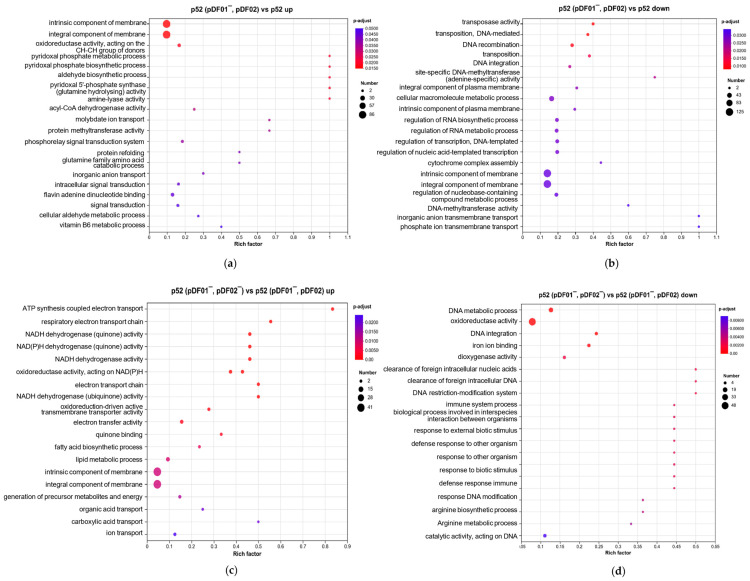
GO enrichment of DEGs in the four comparison groups. (**a**) The upregulated DEGs in group p52 (pDF01^−^, pDF02) vs. p52. (**b**) The downregulated DEGs in group p52 (pDF01^−^, pDF02) vs. p52. (**c**) The upregulated DEGs in group p52 (pDF01^−^, pDF02^−^) vs. p52 (pDF01^−^, pDF02). (**d**) The downregulated DEGs in group p52 (pDF01^−^, pDF02^−^) vs. p52 (pDF01^−^, pDF02).

The specific pathways affected by plasmid loss were analyzed through GO enrichment analysis to further explore the roles of plasmids and determine the regulatory and adaptation mechanisms involved in the loss of pDF01 in strain p52. Compared with those in pDF01-bearing strain p52, a total of 1045 DEGs were identified after pDF01 loss, including 380 upregulated genes and 665 downregulated genes (Figure S9a). A high number of DEGs associated with membrane components were found among upregulated and downregulated genes (Figure 5a,b). The upregulated genes were enriched in processes associated with biosynthetic and metabolic pyridoxal phosphate (PLP), signal transduction, and oxidoreductase activity (Figure 5a). PLP, as the active form of vitamin B6, serves as a coenzyme in a diverse array of enzymatic reactions, especially in the process of amino acid metabolism [59]. The hydrolysis of glutamine plays a crucial role in the PLP synthesis process (GO ID: 0036381), and the corresponding pathways are also upregulated. Previous studies have shown that the genes involved in glutamine synthesis were overexpressed when the gene expression profiles were altered by four different plasmids in *Pseudomonas aeruginosa* PAO1 [13], which may be associated with nitrogen control [60]. In addition, the pathways involved in glutamine family amino acid catabolic processes were upregulated, which indicated the important role of pDF01 in metabolism and suggested that the cells adjust metabolic activity and adapt to the loss of pDF01. In addition, multiple pathways are involved in transcriptional regulation, including RNA biosynthesis and metabolism, DNA-templated transcription, and the metabolism of nucleobase-containing compounds. Plasmids generally encode transcriptional regulators that play critical roles in the regulatory networks of cells, the absence of which might disrupt the existing balance in gene regulation, leading to wide changes in regulatory activity [14]. Similarly, cells may reallocate resources in response to the loss of pDF01, potentially leading to global genomic regulation. The regulation revealed an adaptation response to increased metabolic demands and reduced transcriptional regulation and transposase activities, which suggested that pDF01 participated in the metabolic processes and transcriptional regulatory activities of strain p52.

#### 3.4.2. pDF02 Played Roles in Cellular Energy Production and Defense Responses

A total of 442 DEGs, including 186 upregulated genes and 256 downregulated genes, were found between p52 (pDF01^−^, pDF02) and p52 (pDF01^−^, pDF02^−^) (Figure S9b). The upregulated genes were enriched in processes related to energy production and electron transport, involving ATP synthesis, the respiratory electron transport chain, and the activities of various oxidoreductase enzymes involved in electron transport (Figure 5c). The upregulation of these pathways can be attributed to the increased expression of multiple NADH-quinone oxidoreductase subunit genes. In addition, some upregulated DEGs were involved in the substance transport and lipid metabolic processes (Figure 5c). Besides, pDF02 encodes four AAA family ATPases, which are enzymes associated with diverse cellular activities. More specifically, these ATPases catalyze the hydrolysis of ATP to supply energy for a multitude of cellular functions [61]. A NAD(P)H-binding protein is also encoded by pDF02, which plays a crucial role in energy metabolism and redox reactions [62]. The energy production strategies of the cells were adjusted in response to pDF02 loss, which required the upregulation of the pathways involved in ATP synthesis and energy metabolism. The downregulated genes were enriched in various processes associated with immune and defense responses, such as the clearance of foreign intracellular nucleic acids and DNA, responding to external biotic stimuli and other organisms, and immune system processes (Figure 5d), which can be attributed to the decreased expression of genes encoding restriction endonucleases. Other processes related to DNA metabolism and integration, oxidoreductase activity, and iron ion binding were also downregulated. Restriction endonucleases serve as bacterial defense mechanisms against external DNA [63]. The loss of genes encoding restriction endonucleases on pDF02 resulted in a compromised ability to defend against external genetic challenges, with the downregulation of associated immune response pathways. Based on transcription regulation, energy demands were increased and defense responses were decreased due to the loss of pDF02, highlighting its critical contributions to energy production and cellular defense mechanisms. This result was consistent with the growth variation after pDF02 loss, and revealed that pDF02 carriage was favorable to the growth of strain p52.

## 4. Conclusions

This study highlighted the different roles of catabolic megaplasmids in their native host strain p52 and provided in-depth insights into the potential mechanisms by which bacteria adapt to the stress resulting from plasmid loss. The loss of plasmids resulted in more pronounced impacts on cellular physiology, which were characterized by disparities in growth and biofilm formation. Specifically, pDF02 played an essential role in biofilm formation. Growth analysis revealed that pDF01 was unfavorable to strain p52 growth in contrast to pDF02 under nonselective pressure. However, pDF01 played a predominant role in the degradation pathway, primarily in the initial step of dibenzofuran degradation. The transcriptome analysis revealed a series of enriched pathways, leading to cellular adaptations in response to the loss of the native plasmids. The alterations indicated the essential role of pDF01 in metabolism, transcription, and transposase activities and that of pDF02 in energy production and cellular defense. This study demonstrated that different dioxin-catabolic plasmids exert distinct impacts on the same native host, and different regulatory strategies are adopted by the host in response to the plasmid loss. This study enriches our knowledge of the behavior, survival, and adaptation of bacteria harboring catabolic plasmids in the environment, and provides theoretical knowledge for enhancing plasmid-mediated bioremediation.

## Data Availability

The raw RNA sequencing data are available in the NCBI Sequencing Read Archive (BioProject ID PRJNA1117748). Data are contained within the article.

## Supplementary Materials

The following supporting information can be downloaded at: https://www.mdpi.com/article/10.3390/microorganisms12081700/s1, Materials and Methods: Growth measurement at different dibenzofuran concentrations; Figure S1: Growth of strain p52 on different concentrations of dibenzofuran in the soil; Figure S2: Confirmation of plasmid loss; Figure S3: Specific growth rate of the clones of strain p52, p52 (pDF01^−^, pDF02), and p52 (pDF01^−^, pDF02^−^) without selective pressure; Figure S4: Scanning Electron Microscope (SEM) images of the clones of (a) strain p52, (b) p52 (pDF01^−^, pDF02), and (c) p52 (pDF01^−^, pDF02^−^) at the mid-logarithmic phase; Figure S5: Morphological analysis of the clones of strain p52 at early- and late logarithmic phase on a population level using flow cytometry; Figure S6: Relative expression level of the genes involved in cell division by RNA-seq in the two comparison groups, p52 (pDF01^−^, pDF02) vs. p52, and p52 (pDF01^−^, pDF02^−^) vs. p52; Figure S7: Genetic maps of (a) pDF01 and (b) pDF02; Figure S8: Cluster heat map for the gene expression levels of the strain p52, p52 (pDF01^−^, pDF02), and p52 (pDF01^−^, pDF02^−^); Figure S9: Scatter plots of the differentially expressed genes in strain p52 after plasmid loss. Table S1: The primers used in this study.

## References

[1] Wimmerová S., van den Berg M., Chovancová J., Patayová H., Jusko T.A., van Duursen M.B.M., Palkovičová Murínová Ľ., Canton R.F., van Ede K.I., Trnovec T. (2016). Relative effect potency estimates of dioxin-like activity for dioxins, furans, and dioxin-like PCBs in adults based on cytochrome P450 1A1 and 1B1 gene expression in blood. Environ. Int..

[2] Kirkok S.K., Kibet J.K., Kinyanjui T.K., Okanga F.I. (2020). A review of persistent organic pollutants: Dioxins, furans, and their associated nitrogenated analogues. SN Appl. Sci..

[3] Zheng W., Zhao H., Liu Q., Crabbe M.J.C., Qu W. (2022). Spatial-temporal distribution, cancer risk, and disease burden attributed to the dietary dioxins exposure of Chinese residents. Sci. Total Environ..

[4] Pajurek M., Mikolajczyk S., Warenik-Bany M. (2023). Occurrence and dietary intake of dioxins, furans (PCDD/Fs), PCBs, and flame retardants (PBDEs and HBCDDs) in baby food and infant formula. Sci. Total Environ..

[5] Köstlbacher S., Collingro A., Halter T., Domman D., Horn M. (2021). Coevolving plasmids drive gene flow and genome plasticity in host-associated intracellular bacteria. Curr. Biol..

[6] Kim J., Park W. (2018). Genome analysis of naphthalene-degrading *Pseudomonas* sp. AS1 harboring the megaplasmid pAS1. J. Microbiol. Biotechnol..

[7] Dennis J.J., Zylstra G.J. (2004). Complete sequence and genetic organization of pDTG1, the 83 kilobase naphthalene degradation plasmid from *Pseudomonas putida* strain NCIB 9816-4. J. Mol. Biol..

[8] Shimizu S., Kobayashi H., Masai E., Fukuda M. (2001). Characterization of the 450-kb linear plasmid in a polychlorinated biphenyl degrader, *Rhodococcus* sp. strain RHA1. Appl. Environ. Microbiol..

[9] Maeda K., Nojiri H., Shintani M., Yoshida T., Habe H., Omori T. (2003). Complete nucleotide sequence of carbazole/dioxin-degrading plasmid pCAR1 in *Pseudomonas resinovorans* strain CA10 indicates its mosaicity and the presence of large catabolic transposon Tn*4676*. J. Mol. Biol..

[10] Elken E., Heinaru E., Jõesaar M., Heinaru A. (2020). Formation of new PHE plasmids in pseudomonads in a phenol-polluted environment. Plasmid.

[11] San Millan A., MacLean R.C. (2017). Fitness costs of plasmids: A limit to plasmid transmission. Microbiol. Spectr..

[12] Vial L., Hommais F. (2020). Plasmid-chromosome cross-talks. Environ. Microbiol..

[13] San Millan A., Toll-Riera M., Qi Q., Betts A., Hopkinson R.J., McCullagh J., MacLean R.C. (2018). Integrative analysis of fitness and metabolic effects of plasmids in *Pseudomonas aeruginosa* PAO1. ISME J..

[14] Thompson C.M.A., Hall J.P.J., Chandra G., Martins C., Saalbach G., Panturat S., Bird S.M., Ford S., Little R.H., Piazza A. (2023). Plasmids manipulate bacterial behaviour through translational regulatory crosstalk. PLoS Biol..

[15] Rozkov A., Avignone-Rossa C.A., Ertl P.F., Jones P., O'Kennedy R.D., Smith J.J., Dale J.W., Bushell M.E. (2004). Characterization of the metabolic burden on *Escherichia coli* DH1 cells imposed by the presence of a plasmid containing a gene therapy sequence. Biotechnol. Bioeng..

[16] Diaz Ricci J.C., Hernández M.E. (2000). Plasmid effects on *Escherichia coli* metabolism. Crit. Rev. Biotechnol..

[17] Horemans B., Raes B., Brocatus H., T’Syen J., Rombouts C., Vanhaecke L., Hofkens J., Springael D. (2017). Genetic (in) stability of 2, 6-dichlorobenzamide catabolism in *Aminobacter* sp. strain MSH1 biofilms under carbon starvation conditions. Appl. Environ. Microbiol..

[18] Takahashi Y., Shintani M., Li L., Yamane H., Nojiri H. (2009). Carbazole-degradative IncP-7 plasmid pCAR1.2 is structurally unstable in *Pseudomonas fluorescens* Pf0-1, which accumulates catechol, the intermediate of the carbazole degradation pathway. Appl. Environ. Microbiol..

[19] Ghigo J.M. (2001). Natural conjugative plasmids induce bacterial biofilm development. Nature.

[20] Yao S., Hao L., Zhou R., Jin Y., Huang J., Wu C. (2022). Formation of biofilm by *Tetragenococcus halophilus* benefited stress tolerance and anti-biofilm activity against *S. aureus* and *S. typhimurium*. Front. Microbiol..

[21] Howard S.A., McCarthy R.R. (2023). Modulating biofilm can potentiate activity of novel plastic-degrading enzymes. NPJ Biofilms Microbiomes.

[22] Edwards S.J., Kjellerup B.V. (2013). Applications of biofilms in bioremediation and biotransformation of persistent organic pollutants, pharmaceuticals/personal care products, and heavy metals. Appl. Microbiol. Biotechnol..

[23] Fan L.P., Yu Y., Huang S., Liao W., Huang Q.S., Du F.L., Xiang T.X., Wei D.D., Wan L.G., Zhang W. (2022). Genetic characterization and passage instability of a novel hybrid virulence plasmid in a ST23 hypervirulent *Klebsiella pneumoniae*. Front. Cell Infect. Microbiol..

[24] Michael V., Frank O., Bartling P., Scheuner C., Göker M., Brinkmann H., Petersen J. (2016). Biofilm plasmids with a rhamnose operon are widely distributed determinants of the ‘swim-or-stick’ lifestyle in roseobacters. ISME J..

[25] Coulson G.B., Miranda-CasoLuengo A.A., Miranda-CasoLuengo R., Wang X., Oliver J., Willingham-Lane J.M., Meijer W.G., Hondalus M.K. (2015). Transcriptome reprogramming by plasmid-encoded transcriptional regulators is required for host niche adaption of a macrophage pathogen. Infect. Immun..

[26] Dineen R.L., Bottacini F., O’Connell-Motherway M., van Sinderen D. (2024). Transcriptional landscape of the pMP7017 megaplasmid and its impact on the *Bifidobacterium breve* UCC2003 transcriptome. Microb. Biotechnol..

[27] Shintani M., Takahashi Y., Tokumaru H., Kadota K., Hara H., Miyakoshi M., Naito K., Yamane H., Nishida H., Nojiri H. (2010). Response of the *Pseudomonas* host chromosomal transcriptome to carriage of the IncP-7 plasmid pCAR1. Environ. Microbiol..

[28] Peng P., Yang H., Jia R., Li L. (2013). Biodegradation of dioxin by a newly isolated *Rhodococcus* sp. with the involvement of self-transmissible plasmids. Appl. Microbiol. Biotechnol..

[29] Ren C., Wang Y., Tian L., Chen M., Sun J., Li L. (2018). Genetic bioaugmentation of activated sludge with dioxin-catabolic plasmids harbored by *Rhodococcus* sp. Strain p52. Environ. Sci. Technol..

[30] Wu Y., Wang X., Zhao W., Wang X., Yang Z., Li L. (2024). Roles of n-hexadecane in the degradation of dibenzofuran by a biosurfactant-producing bacterium *Rhodococcus* sp. J. Clean. Prod..

[31] Wang X., Wu Y., Fu C., Zhao W., Li L. (2024). Metabolic cross-feeding between the competent degrader *Rhodococcus* sp. strain p52 and an incompetent partner during catabolism of dibenzofuran: Understanding the leading and supporting roles. J. Hazard. Mater..

[32] Sambrook J., Fritsch E.F., Maniatis T. (1989). Molecular Cloning: A Laboratory Manual.

[33] Li L., Li Q., Li F., Shi Q., Yu B., Liu F., Xu P. (2006). Degradation of carbazole and its derivatives by a *Pseudomonas* sp. Appl. Microbiol. Biotechnol..

[34] Ghosh S., Mahapatra N.R., Ramamurthy T., Banerjee P.C. (2000). Plasmid curing from an acidophilic bacterium of the genus *Acidocella*. FEMS Microbiol. Lett..

[35] Ka J.O., Tiedje J.M. (1994). Integration and excision of a 2,4-dichlorophenoxyacetic acid-degradative plasmid in *Alcaligenes paradoxus* and evidence of its natural intergeneric transfer. J. Bacteriol..

[36] Prieto G.A., Snigdha S., Baglietto-Vargas D., Smith E.D., Berchtold N.C., Tong L., Ajami D., LaFerla F.M., Rebek J., Cotman C.W. (2015). Synapse-specific IL-1 receptor subunit reconfiguration augments vulnerability to IL-1β in the aged hippocampus. Proc. Natl. Acad. Sci. USA.

[37] Chen C.H., Puliafito A., Cox B.D., Primo L., Fang Y., Di Talia S., Poss K.D. (2016). Multicolor cell barcoding technology for long-term surveillance of epithelial regeneration in zebrafish. Dev. Cell.

[38] Stepanović S., Vuković D., Dakić I., Savić B., Švabić-Vlahović M. (2000). A modified microtiter-plate test for quantification of staphylococcal biofilm formation. J. Microbiol. Methods.

[39] Schmittgen T.D., Livak K.J. (2008). Analyzing real-time PCR data by the comparative C_T_ method. Nat. Protoc..

[40] Lili L.N., Britton N.F., Feil E.J. (2007). The persistence of parasitic plasmids. Genetics.

[41] Nojiri H. (2012). Structural and molecular genetic analyses of the bacterial carbazole degradation system. Biosci. Biotechnol. Biochem..

[42] Enne V.I., Bennett P.M., Livermore D.M., Hall L.M. (2004). Enhancement of host fitness by the *sul2*-coding plasmid p9123 in the absence of selective pressure. J. Antimicrob. Chemother..

[43] Marteyn B.S., Karimova G., Fenton A.K., Gazi A.D., West N., Touqui L., Prevost M.C., Betton J.M., Poyraz O., Ladant D. (2014). ZapE is a novel cell division protein interacting with FtsZ and modulating the Z-ring dynamics. mBio.

[44] Schubert K., Sieger B., Meyer F., Giacomelli G., Böhm K., Rieblinger A., Lindenthal L., Sachs N., Wanner G., Bramkamp M. (2017). The antituberculosis drug ethambutol selectively blocks apical growth in CMN group bacteria. mBio.

[45] Taguchi A., Welsh M.A., Marmont L.S., Lee W., Sjodt M., Kruse A.C., Kahne D., Bernhardt T.G., Walker S. (2019). FtsW is a peptidoglycan polymerase that is functional only in complex with its cognate penicillin-binding protein. Nat. Microbiol..

[46] Aly H.A., Huu N.B., Wray V., Junca H., Pieper D.H. (2008). Two angular dioxygenases contribute to the metabolic versatility of dibenzofuran-degrading *Rhodococcus* sp. strain HA01. Appl. Environ. Microbiol..

[47] Nojiri H., Kamakura M., Urata M., Tanaka T., Chung J.S., Takemura T., Yoshida T., Habe H., Omori T. (2002). Dioxin catabolic genes are dispersed on the *Terrabacter* sp. DBF63 genome. Biochem. Biophys. Res. Commun..

[48] Kasuga K., Nitta A., Kobayashi M., Habe H., Nojiri H., Yamane H., Omori T., Kojima I. (2013). Cloning of *dfdA* genes from *Terrabacter* sp. strain DBF63 encoding dibenzofuran 4,4a-dioxygenase and heterologous expression in *Streptomyces lividans*. Appl. Microbiol. Biotechnol..

[49] Carrilero L., Kottara A., Guymer D., Harrison E., Hall J.P.J., Brockhurst M.A. (2021). Positive selection inhibits plasmid coexistence in bacterial genomes. mBio.

[50] Hall J.P.J., Harrison E., Lilley A.K., Paterson S., Spiers A.J., Brockhurst M.A. (2015). Environmentally co-occurring mercury resistance plasmids are genetically and phenotypically diverse and confer variable context-dependent fitness effects. Environ. Microbiol..

[51] Singh A., Ahmed A., Prasad K.N., Khanduja S., Singh S.K., Srivastava J.K., Gajbhiye N.S. (2015). Antibiofilm and membrane-damaging potential of cuprous oxide nanoparticles against *Staphylococcus aureus* with reduced susceptibility to vancomycin. Antimicrob. Agents Chemother..

[52] Moye Z.D., Gormley C.M., Davey M.E. (2019). Galactose impacts the size and intracellular composition of the asaccharolytic oral pathobiont *Porphyromonas gingivalis*. Appl. Environ. Microbiol..

[53] Chai Y., Beauregard P.B., Vlamakis H., Losick R., Kolter R. (2012). Galactose metabolism plays a crucial role in biofilm formation by *Bacillus subtilis*. mBio.

[54] Barreto M., Jedlicki E., Holmes D.S. (2005). Identification of a gene cluster for the formation of extracellular polysaccharide precursors in the chemolithoautotroph *Acidithiobacillus ferrooxidans*. Appl. Environ. Microbiol..

[55] Ha D.G., O'Toole G.A. (2015). c-di-GMP and its effects on biofilm formation and dispersion: A *Pseudomonas aeruginosa* review. Microbial Spectr..

[56] Guo Y., Sagaram U.S., Kim J.S., Wang N. (2010). Requirement of the *galU* gene for polysaccharide production by and pathogenicity and growth *in planta* of *Xanthomonas citri* subsp. citri. Appl. Environ. Microbiol..

[57] Billane K., Harrison E., Cameron D., Brockhurst M.A. (2022). Why do plasmids manipulate the expression of bacterial phenotypes?. Philos. Trans. R. Soc. Lond. B Biol. Sci..

[58] Lang K.S., Johnson T.J. (2015). Transcriptome modulations due to A/C2 plasmid acquisition. Plasmid.

[59] Wu M., Crismaru C.G., Salo O., Bovenberg R.A., Driessen A.J. (2020). Impact of classical strain improvement of *Penicillium rubens* on amino acid metabolism during β-lactam production. Appl. Environ. Microbiol..

[60] Janssen D.B., Herst P.M., Joosten H.M., van der Drift C. (1981). Nitrogen control in *Pseudomonas aeruginosa*: A role for glutamine in the regulation of the synthesis of NADP-dependent glutamate dehydrogenase, urease and histidase. Arch. Microbiol..

[61] Khan Y.A., White K.I., Brunger A.T. (2022). The AAA+ superfamily: A review of the structural and mechanistic principles of these molecular machines. Crit. Rev. Biochem. Mol. Biol..

[62] Blacker T.S., Duchen M.R., Bain A.J. (2023). NAD(P)H binding configurations revealed by time-resolved fluorescence and two-photon absorption. Biophys. J..

[63] Loenen W.A., Dryden D.T., Raleigh E.A., Wilson G.G., Murray N.E. (2014). Highlights of the DNA cutters: A short history of the restriction enzymes. Nucleic Acids Res..

