# Supplementary material for: Different Roles of Dioxin-Catabolic Plasmids in Growth, Biofilm Formation, and Metabolism of Rhodococcus sp. Strain p52
Source: Microorganisms. 2024 Aug 17;12(8):1700. doi: 10.3390/microorganisms12081700 (PMC11357670; doi:10.3390/microorganisms12081700)
Supplement: Supplementary file 1 [file microorganisms-12-01700-s001.zip › microorganisms-3113736-supplementary.pdf]

Supplemental material

Different roles of dioxin-catabolic plasmids in growth, biofilm formation, and metabolism of *Rhodococcus* sp. strain p52

Xu Wang [1], Yanan Wu [1], Meng Chen[1,2], Changai Fu[1], Hangzhou Xu[1] and Li Li [1,*]

[1] Shandong Provincial Key Laboratory of Water Pollution Control and Resource Reuse, School of Environmental Science and Engineering, Shandong University, 72 Binhai Road, Qingdao, China, 266237
[2] Marine Genomics and Biotechnology Program, Institute of Marine Science and Technology, Shandong University, 72 Binhai Road, Qingdao, China, 266237
* Correspondence: lili@sdu.edu.cn;

Supplemental material Content

Materials and Methods: Growth measurement at different dibenzofuran concentrations.

Figure S1: Growth of strain p52 on different concentrations of dibenzofuran in the soil;

Figure S2: Confirmation of plasmid loss;

Figure S3: Specific growth rate of the clones of strain p52, p52 (pDF01^{-}, pDF02), and p52 (pDF01^{-}, pDF02^{-}) without selective pressure;

Figure S4: Scanning Electron Microscope (SEM) images of the clones of (a) strain p52, (b) p52 (pDF01^{-}, pDF02), and (c) p52 (pDF01^{-}, pDF02^{-}) at the mid-logarithmic phase;

Figure S5: Morphological analysis of the clones of strain p52 at early and late logarithmic phase on a population level using flow cytometry;

Figure S6: Relative expression level of the genes involved in cell division by RNA-seq in the two comparison groups, p52 (pDF01^{-}, pDF02) vs p52, and p52 (pDF01^{-}, pDF02^{-}) vs p52;

Figure S7: Genetic maps of (a) pDF01 and (b) pDF02;

Figure S8: Cluster heat map for the gene expression levels of the strain p52, p52 (pDF01^{-}, pDF02), and p52 (pDF01^{-}, pDF02^{-});

Figure S9: Scatter plots of the differentially expressed genes in strain p52 after plasmid loss.

Table S1: The primers used in this study.

Measurement of strain p52 *Growth on different concentrations of dibenzofuran*

The growth of strain p52 on dibenzofuran at different concentrations were evaluated using 250 mL Erlenmeyer flasks, each containing 50 g (dry weight) of sterilized soil that had been sieved through a 2 mm mesh. Dibenzofuran stock solution was mixed with water and sprayed to the soil samples at concentrations of 0 mg/kg, 100 mg/kg, and 1000 mg/kg (soil). Then, strain p52 was inoculated into the soil to achieve a final concentration of 10^6 to 10^7 CFU/g (soil). During the experiment, soil samples were collected, and soil suspensions were prepared. The suspensions were serially diluted and spread onto dibenzofuran-supplemented agar plate to assess bacterial growth. Three replicates were set up for each sample.

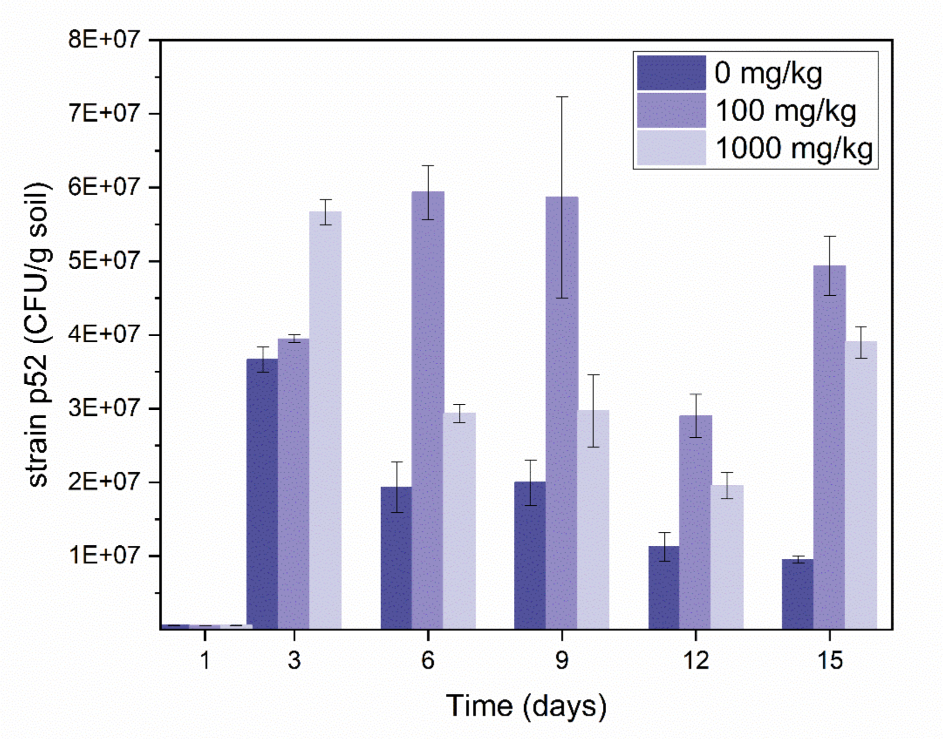

Figure S1. Growth of strain p52 on different concentrations of dibenzofuran in the soil.

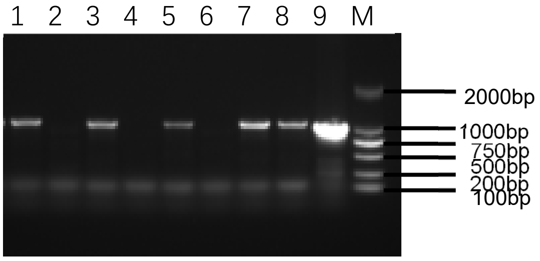

(a)

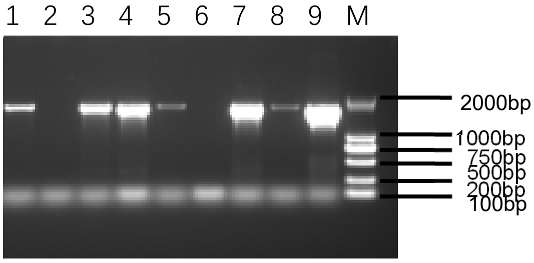

(b)

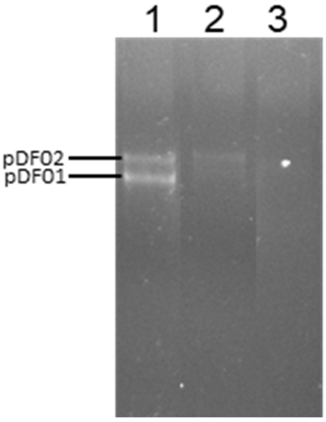

(c)

Figure S2. Confirmation of plasmid loss. (a) Electrophoresis analysis of the amplification product targeting the angular dioxygenase gene *dfdA* (1.06 kb) located on pDF01. (b) Electrophoresis analysis of the amplification product targeting the angular dioxygenase gene *dbfA* (1.84 kb) located on pDF02. DNA Maker is shown in Lane M, and Lane 9 is the amplification result of plasmid-bearing clone of p52 as a positive control. The detected clones are shown in Lanes 1–8. (c) Electrophoresis analysis of plasmid DNA extracted from strain p52, p52 (pDF01^{-}, pDF02), and p52 (pDF01^{-}, pDF02^{-}). Lanes 1, 2, 3 correspond to strain p52, p52 (pDF01^{-}, pDF02), and p52 (pDF01^{-}, pDF02^{-}), respectively.

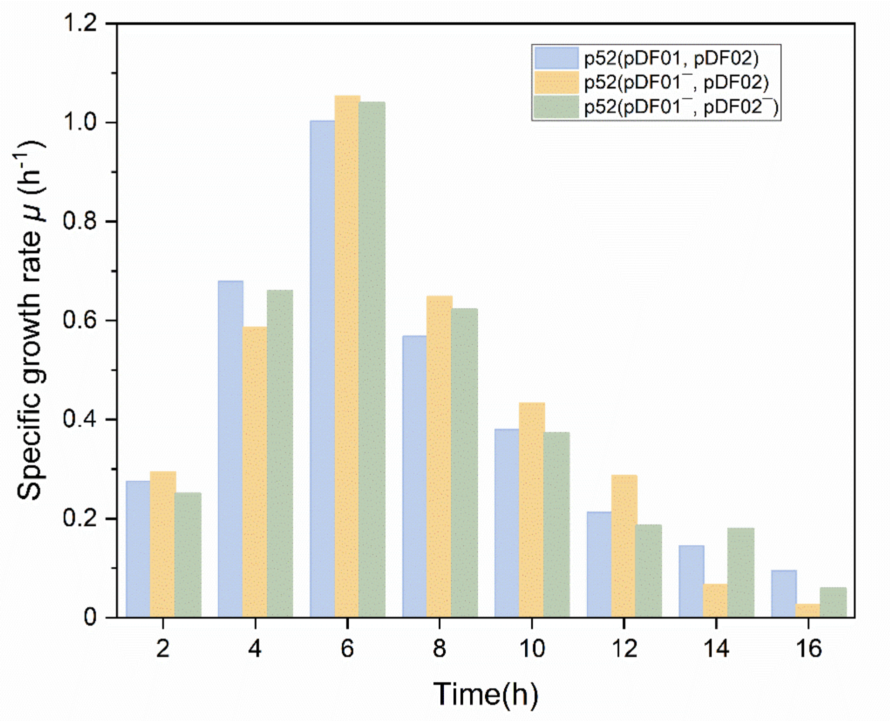

Figure S3. Specific growth rate of the clones of strain p52, p52 (pDF01$^-$, pDF02), and p52 (pDF01$^-$, pDF02$^-$) without selective pressure.

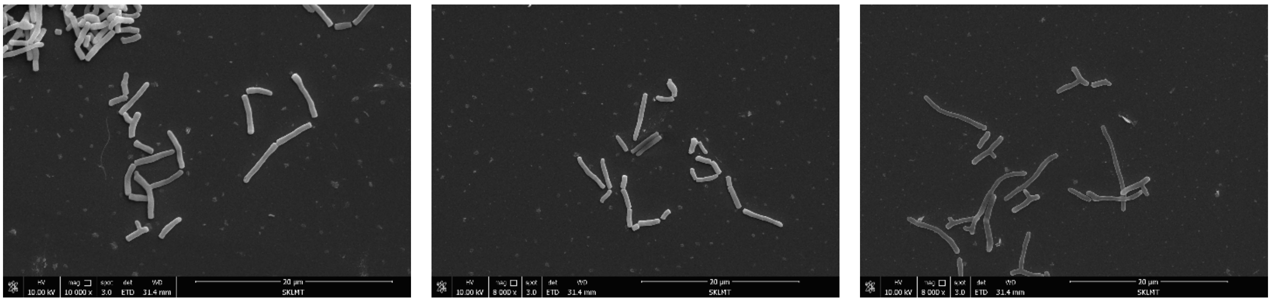

(a) (b) (c)

Figure S4. SEM images of the clones of (a) strain p52, (b) p52 (pDF01⁻, pDF02), and (c) p52 (pDF01⁻, pDF02⁻) at the mid-logarithmic phase.

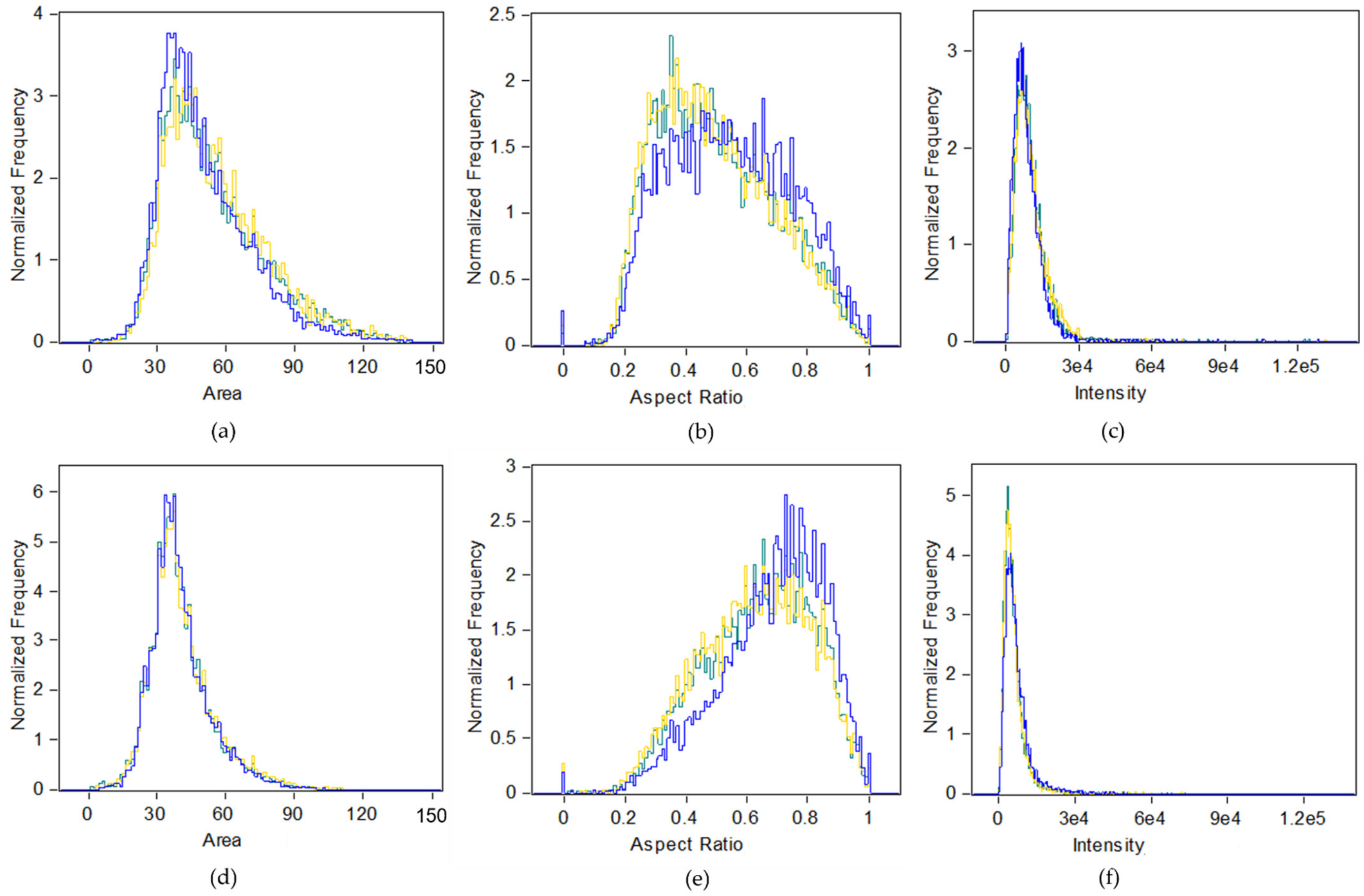

Figure S5. Morphological analysis of the clones of strain p52 at early and late logarithmic phase on a population level using flow cytometry. The clones of strain p52, p52 (pDF01^{-}, pDF02) and p52 (pDF01^{-}, pDF02^{-}) are in blue, yellow, and green lines, respectively. The upper three figures represent the strains in the early logarithmic phase (OD_{600} = 0.5) (a-c), while the lower three figures represent the strains in the late logarithmic phase (OD_{600} = 1.7) (d-f). The normalized frequency of forward scatter area to estimate cell size is shown in (a)&(d). The normalized frequency of aspect ratio to estimate cell shape is shown in (b)&(e). The normalized frequency of side scatter intensity to estimate cellular granularity is shown in (c)&(f).

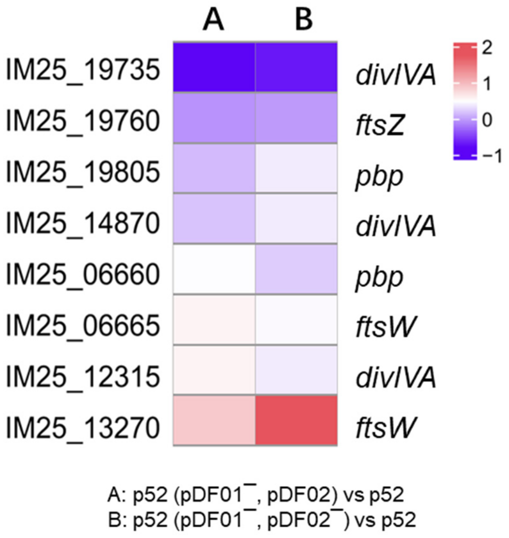

Figure S6. Relative expression level of the genes involved in cell division by RNA-seq in the two comparison groups, p52 (pDF01$^-$, pDF02) vs p52, and p52 (pDF01$^-$, pDF02$^-$) vs p52. The gene ID and name are listed on the left and right, respectively. The relative expression level is expressed as $\log_2$(fold change) and reflected by the color as labeled on the top right.

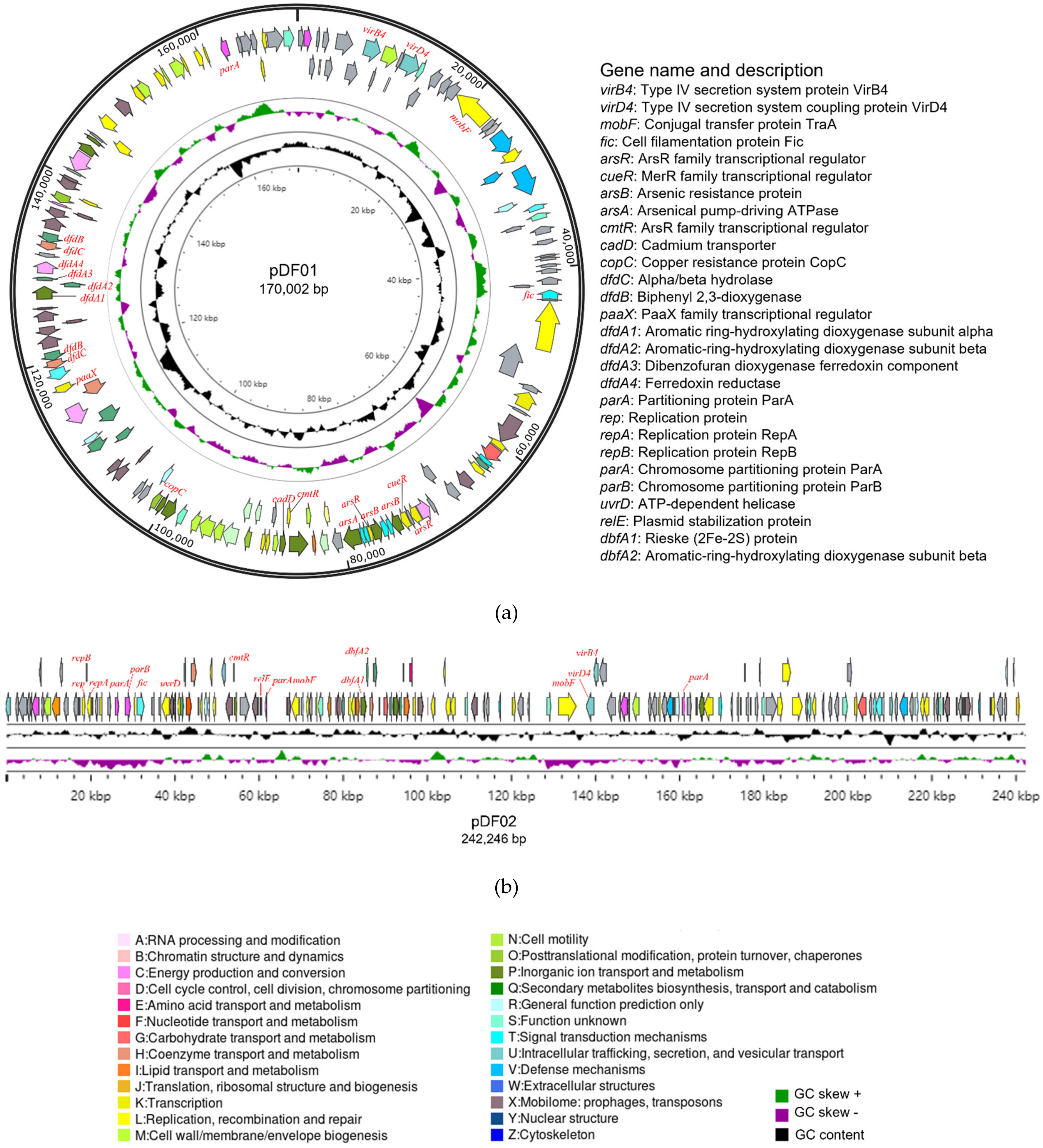

Figure S7. Genetic maps of (a) pDF01 and (b) pDF02, with genes assigned to COG categories and depicted in different colors. The names and descriptions of partial genes are shown on the right. Rings inside represent GC content (black) and GC skew (green and purple). The COG category assignments are present at the bottom of the figure.

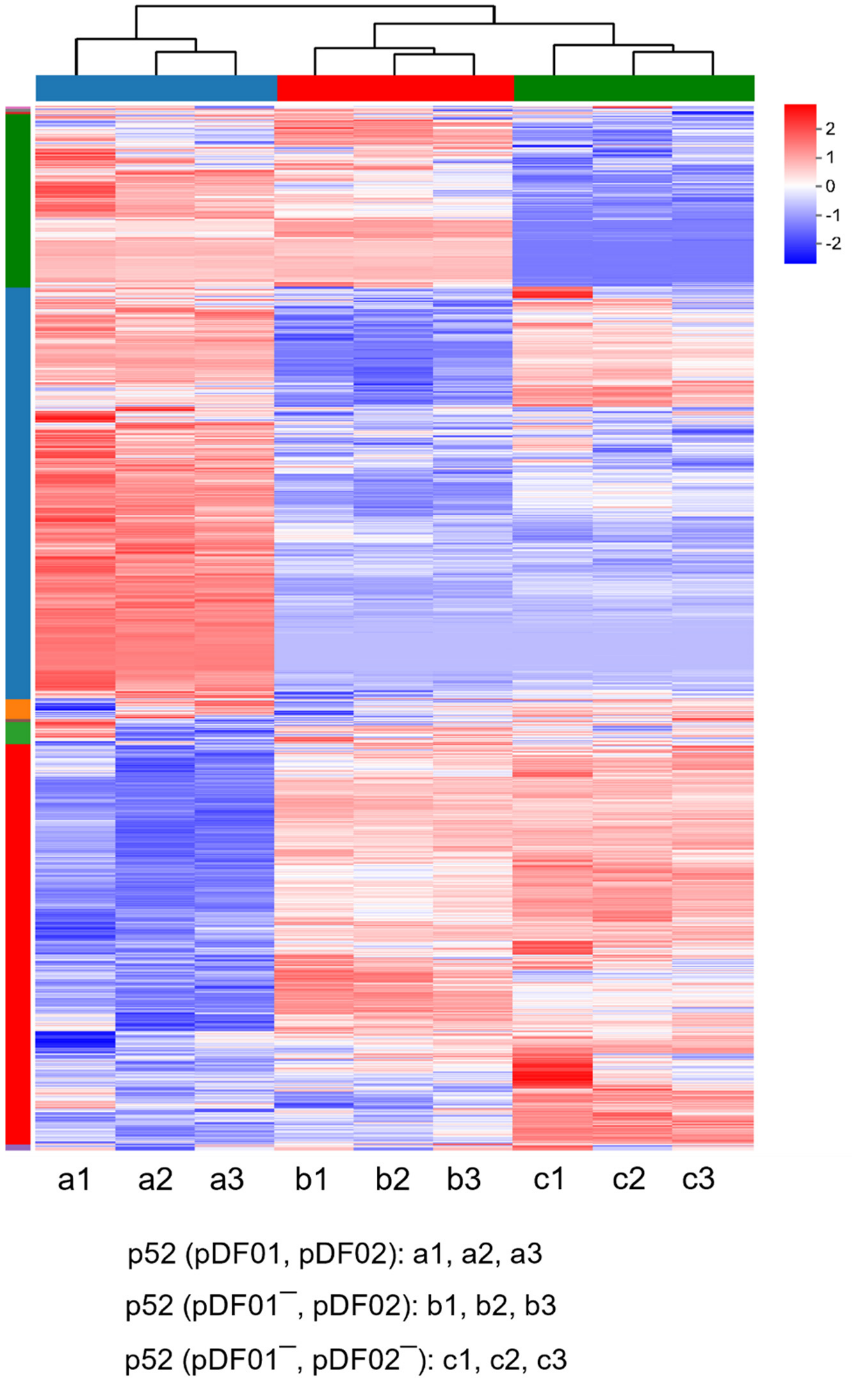

Figure S8. Cluster heat map for the gene expression levels of the strain p52, p52 (pDF01$^-$, pDF02), and p52 (pDF01$^-$, pDF02$^-$). Each sample was replicated three times biologically. The color represents the gene expression levels through normalizing the data. Logarithmic transformation was applied to (TPM+1), followed by Z-score standardization.

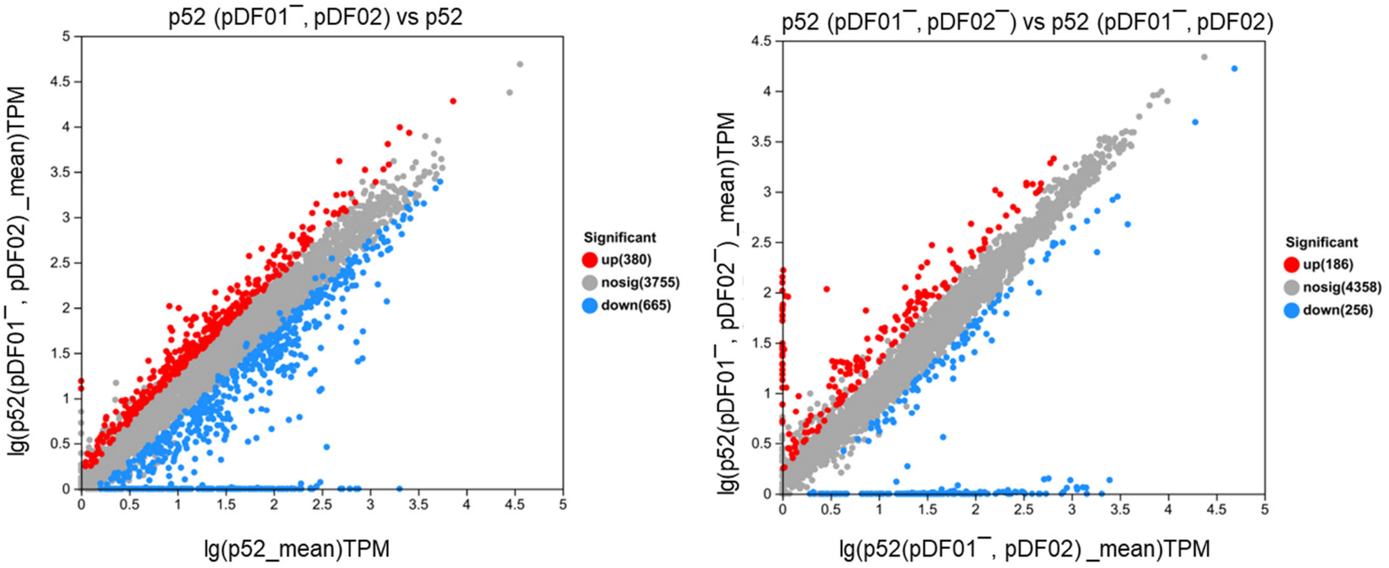

(a) (b)

Figure S9. Scatter plots of the differentially expressed genes in strain p52 after plasmid loss. The DEGs in group p52 (pDF01^{-}, pDF02) vs p52 is shown in (a) and that in group p52 (pDF01^{-}, pDF02^{-}) vs p52 (pDF01^{-}, pDF02) is shown in (b).

Table S1. The primers used in this study.

Targets	Oligonucleotide sequence (5′ to 3′)	Amplicon size (bp)	Location
**Primers used for PCR**			
dfdA	F: GCAGTCTGTACCGACGCT<br>R: GAGTGCGACGGGATGGAC	1061	pDF01
dbfA	F: GCTCATGACCAGCATTAGCG<br>R: GGGCCTCAGAAGAAGATGGAG	1841	pDF02
**Primers used for qPCR**			
Reference gene			
rpoB	F: TGGGTGACTTCCCGATGA<br>R: TGACCTTGACGCTGTGC	153	chromosome
Dibenzofuran degradation genes on pDF01 and pDF02			
dfdA1	F: CTTCTTCTCGACCTACAT<br>R: GGCAGACCTTCATCCCAC	110	pDF01
dfdA2	F: CGACGACGACAAACCGAA<br>R: CGATAGACGAGGAAGTGGG	171	pDF01
dfdA3	F: GGACGAAGGTCTGTGCC<br>R: ACGAGTGCGACGGGAT	234	pDF01
dfdA4	F: CTTCGTCAGTCCGTTCAC<br>R: TGGAGCCGATGTCCGTAT	156	pDF01
dfdB	F: GGCGAGAAGACGACCAGC<br>R: CGAAGCCAGCACTACCAA	163	pDF01
dfdC	F: CGAAGGGCTGAAAGTGA<br>R: ATGCCACGGACGGTAT	228	pDF01
IM25_24385	F: TGGGTGACTTCCCGATGA<br>R: TGACCTTGACGCTGTGC	153	pDF01
IM25_24375	F: TCCGGCACTCTCTCTGCTTT<br>R: TTTCATCACGGGTCGCTGG	106	pDF01
IM25_24380	F: CTGACCGGCTACACCGAC<br>R: ACACGATCTTTCCCGGTGTG	136	pDF01
dbfA1	F: AGGCGATCGAGTACGTCAAC<br>R: ATTGGAAGAGCCCGAAGACG	153	pDF02
dbfA2	F: CCAAGCGGATGGAGCG<br>R: GATGGCACGGGGCAAC	272	pDF02
IM25_23265	F: GTGGTGGAGATTGGCCTCTT<br>R: GGTCCACTGCTCCTTCATCC	180	pDF02
IM25_23270	F: TCATTACATCATGCCCGTCCG<br>R: ACGATGATTTCGTCGGGGTT	219	pDF02
Genes associated with biofilm formation			
galU	F: AAGAAGAAGCAGGAGTGCGTAA<br>R: GTTCGATGCCCGGCGTGT	135	chromosome
pgm	F: GTTCGATGCCCGGCGTGT<br>R: AGCAGGGCGAGGACGA	226	chromosome
galE	F: CGACGTTGAAGTACCGCAGAC<br>R: TACGGCGAACCCGACAGC	154	chromosome
dgc	F: TGCTGGGTCGTCTCGAAGTG<br>R: GCATCTGCTGCTGGTGGTG	210	chromosome
galT	F: CGAAGGTGACCCACTCCC	156	chromosome

	R: GCCGGGAACTGATCTATT		
pde	F: CGTGGTGTACCGCGGAGT<br>R: CGCAGACGCACAAGTAGTTTCA	229	chromosome
galK	F: GCCCTGCCCGCAACAACT<br>R: GTGAGGTCGAAGACGCCA	159	chromosome
